# Identification of a Rare Germline Heterozygous Deletion Involving the Polycistronic miR-17–92 Cluster in Two First-Degree Relatives from a BRCA 1/2 Negative Chilean Family with Familial Breast Cancer: Possible Functional Implications

**DOI:** 10.3390/ijms19010321

**Published:** 2018-01-22

**Authors:** Tomás de Mayo, Annemarie Ziegler, Sebastián Morales, Lilian Jara

**Affiliations:** 1 Center for Genetics and Genomics, School of Medicine, Clinica Alemana Universidad del Desarrollo, Avenida Las Condes 12438 Lo Barnechea, 7710162 Santiago, Chile; tdemayog@udd.cl (T.d.M.); aziegler@udd.cl (A.Z.); 2 Human Genetics Program, Institute of Biomedical Sciences (ICBM), School of Medicine, University of Chile, Avenida Independencia 1027, 8380453 Santiago, Chile; seba.morales.p@gmail.com

**Keywords:** familial breast cancer, miR-17–92 polycistronic cluster, deletion, Chilean population

## Abstract

Micro-RNAs (miRNAs) have emerged as novel gene expression regulators. Recent evidence strongly suggests a role for miRNAs in a large variety of cancer-related pathways. Different studies have shown that 18.7 to 37% of all human miRNA genes are clustered. miR-17–92 polycistronic cluster overexpression is associated with human hematolymphoid and solid malignancies including breast cancer (BC). Here, we report the identification of rs770419845, a rare 6 bp deletion located within the polycistronic miR-17–92 cluster, in two first-degree relatives from a Chilean family with familial BC and negative for point mutations in *BRCA 1/2* genes. The deletion was identified by Sanger sequencing when 99 *BRCA1/2* mutation-negative BC cases with a strong family history were initially screened. In silico analysis predicts that rs770419845 affects the secondary structure and stability of the pre-miR-17–pre-miR-18 region and the entire 17–92 cluster. The deletion was screened in 458 high-risk *BRCA1/2*-negative Chilean families and 480 controls. rs770419845 was not detected in any control but identified in a single family with two cases of BC and other cancers. Both BC cases, the mother and her daughter, carried the deletion. Based on bioinformatic analyses, the location of the deletion and its low frequency, we presume rs770419845 may be a pathogenic variant. Functional studies are needed to support this hypothesis.

## 1. Introduction

Breast cancer (BC) is the most common cancer among women worldwide and has the highest mortality rate among cancers in Chile (15.5/100,000 women) [1]. Genetic factors contribute significantly to BC. Currently, there is consensus that *BRCA1/2* mutations are responsible for 16% of familial BC risk on average [2], leaving much of the genetic component of familial BC unknown [3]. Therefore, it has been proposed that other susceptibility alleles could be responsible for a significant percentage of BC in *BRCA1/2*-negative families. Research to further elucidate the genetic etiology of BC is ongoing, with an emerging interest in epigenetics and gene regulation. A recently discovered gene regulation mechanism involves microRNAs (miRNAs) [4]. miRNAs are single-stranded RNAs that can regulate gene expression by degrading or blocking translation of target mRNAs [5]. Approximately 30% of human genes are regulated by miRNAs [6]. Genome-wide miRNA expression profiling has shown that nearly all types of cancer have a specific profile of up- and downregulated miRNA, attributable to miRNA gene mutations or variations [7].

One of the most remarkable features of animal miRNAs is that they are enriched in clusters [8]. Of the 1881 human miRNA genes annotated to date, studies have shown that 18–37% are clustered [9,10]. The polycistronic miR-17–92 cluster is among the most studied microRNA clusters, mapping to human chromosome 13q.31.3 and encoding for six individual miRNAs [11,12]. First described in hematolymphoid neoplasms [13], the cluster was subsequently reported in several human malignant solid tumors [11,12].

Here, we report the identification of a rare 6 bp germline deletion involving the miR-17–92 cluster in a *BRCA1/2*-negative Chilean family with a history of BC.

## 2. Results

**Figure 1:**
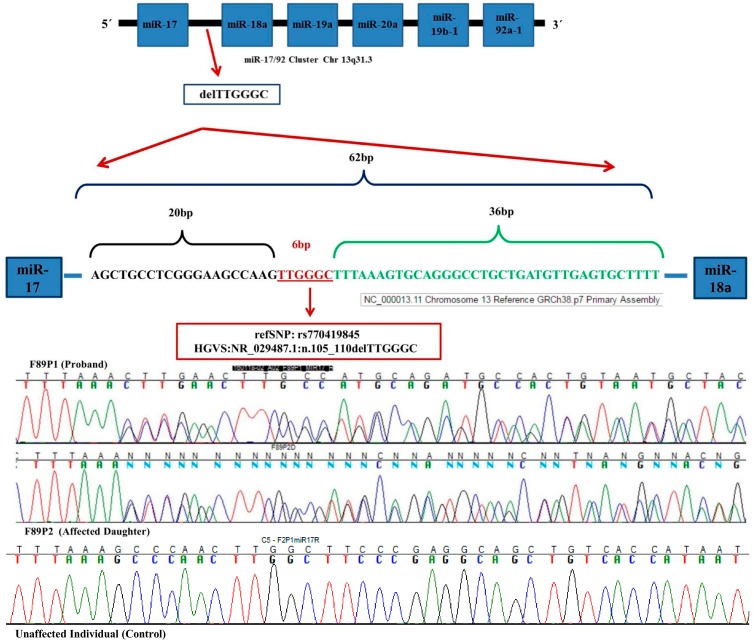
Location, genomic context, and identification by Sanger Sequencing of delTTGGGC (rs770419845) in the polycistronic miR-17–92 cluster. Partial electropherogram showing the deletion in both the proband (F89P1) and her daughter (F89P2). The overlapping peaks occur because both affected individuals are heterozygous for the variant. At the bottom, partial electropherogram of an individual without the deletion (normal homozygous).

**Figure 2:**
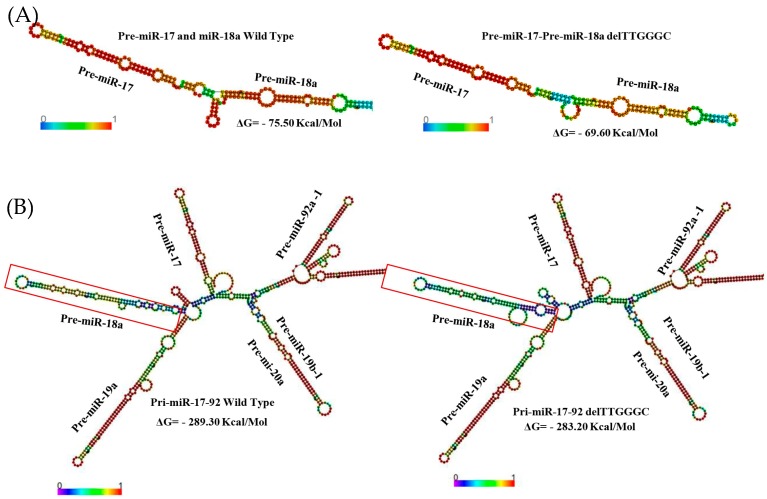
(**A**) Secondary RNA structure predictions for wild-type and delTTGGGC pre-miR-17–pre-miR-18 sequences, using an RNAfold algorithm. Color bars represent base-pair probabilities. (**B**) Secondary RNA structure predictions for wild-type and delTTGGGC polycistronic pri-miR 17–92 cluster sequences, using an RNAfold algorithm. Color bars represent base-pair probabilities. Red boxes depict changes in pre-miR-18a hairpin.

We sequenced the complete pre-miR-17 coding sequence and boundaries in 99 probands from BC families negative for *BRCA1/2* point mutations, to identify pre-miR-17 sequence variations in a Chilean population. We identified only one variant sequence, named rs770419845. This 6 bp deletion (delTTGGGC) is located within the polycistronic miR-17–92 cluster, encoding for six miRNAs in the following order: 5′-miR-17–miR-18a–miR19a–miR-20a–miR-19b-1–miR-92a-1-3′ [11,12]. The deletion is located between the miR-17 and miR-18a sequences—specifically, 20 bp downstream from miR-17 and 36 bp upstream from miR-18a (Figure 1). We used RNAfold algorithm to predict the effect of this deletion on the structure of pre-miR-17, pre-miR-18a, and the miR-17–92 cluster [14]. The deletion produced a change in the secondary structure of the pre-miR-17–pre-miR-18a sequence, decreasing stability (Figure 2A). The deletion also produced a change in the secondary structure and stability of the miR-17–92 cluster (Figure 2B). The hairpin structure most strongly affected by the variant was pre-miR-18. According to the algorithm, the deletion is located in a region of the pri-miR sequence where the base-pairing probability is 1 and the positional entropy tends to zero (both depicted in red) (Figure 2B).

**Figure A1:**
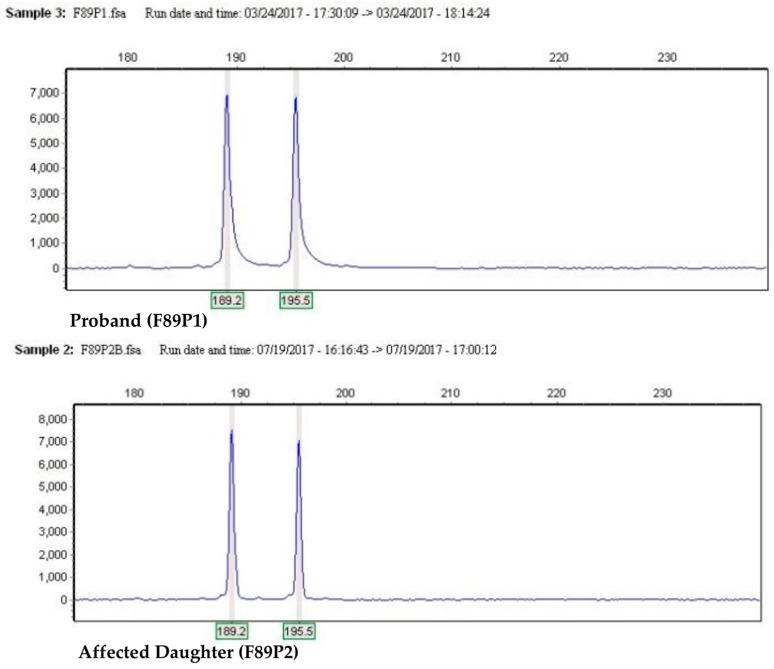
DNA fragment analysis by capillary electrophoresis. The image shows the double peaks of the two affected individuals from the same family (the index case and her daughter), meaning they were heterozygous carriers of the deletion. Note the 6 bp difference between the first peak (189 bp) and the second (195 bp) in both cases.

**Figure A2:**
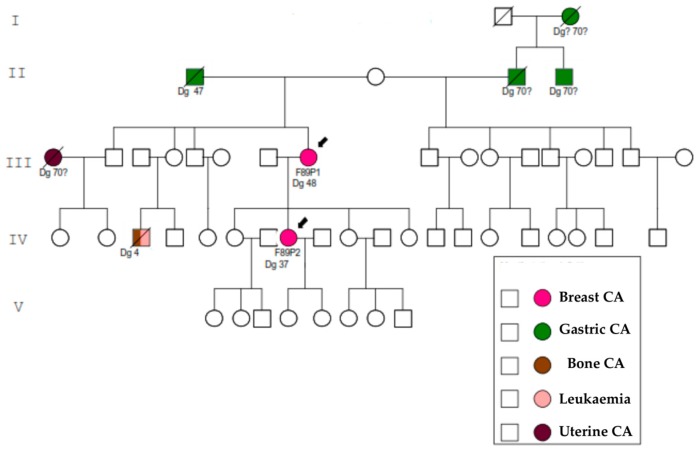
Pedigree of the affected family. Arrows indicate both the proband and her daughter. Roman numbers = Generations.

The variant was analyzed in 500 BC cases from 458 high-risk *BRCA1/2*-negative Chilean families and 480 controls. The deletion was detected in a single family (Figure A1) that meets the criteria for a high-risk family, as there were two early-onset BC cases, four gastric cancer cases, one leukemia case, and one bone cancer case in the family (Figure A2). The index case in this family was diagnosed with invasive left BC at age 48 with positive axillary lymph nodes. The second case was the index case’s daughter, with invasive right BC diagnosed at age 37, nine positive axillary lymph nodes, and in situ carcinoma in the left breast. Both mother and daughter carried the TTGGGC deletion. Therefore, the frequency of the deletion in the BC cohort was 0.4% (2/500). Considering that (a) the deletion affected the secondary structure and stability of the pre-miR-17–pre-miR-18 region and the entire cluster, (b) the deletion was not present in any of the 480 controls, and (c) rs770419845 is a rare variant in *BRCA1/2*-negative BC, this indel is likely pathogenic.

## 3. Discussion

*BRCA1/2* mutations are associated with BC and ovarian cancer OC susceptibility. At present, however, those mutations account for only a minority of familial cases. Therefore, intensive research to identify additional targets is ongoing. It has been proposed that other susceptibility alleles could be responsible for a significant percentage of BC in *BRCA1/2*-negative families. Candidates include pre-microRNAs (pre-miRNAs) or microRNAs (miRNAs), as miRNAs can regulate gene expression by degrading or blocking translation of mRNA targets. Therefore, a *BRCA1/2*-negative individual could have a risk profile equivalent to a *BRCA1/2*-positive individual if the BRCA1/2 mRNA is degraded by a specific mutated miRNA. Thus, pathogenic mutations in microRNA may be implicated in the genetic etiology of some *BRCA1/2*-negative familial BC cases.

miRNAs are involved in many molecular and biological pathways [15]. Variations such as single nucleotide polymorphisms (SNPs) or indels in miRNA gene regions can affect miRNA function by modulating pri-miRNA and pre-miRNA transcription, processing, and maturation, as well as miRNA–mRNA interaction, potentially contributing to cancer susceptibility [16]. Moreover, genetic variability is ethnicity-specific. To date, familial BC-related miRNA variations have not been studied in Latin American populations. In the present study, we searched for pre-miR-17 sequence variants in Chilean *BRCA1/2*-negative familial BC cases.

We identified a single 6 bp indel (delTTGGGC), located within the polycistronic miR-17–92 cluster. This deletion has previously been described only once, by the Exome Aggregation Consortium (ExAC), in 2016 [17]. ExAC comprises a cohort of 60,706 individuals, including healthy individuals (3.04%) and patients with various pathologies, including cancer. The cohort includes individuals of many ethnicities: African/African-American (8.57%), Latino (9.53%), East-Asian (7.2%), Finnish (5.44%), non-Finnish European (54.96%), South Asian (13.59%), and others (0.74%). ExAC detected rs770419845 (Available online: https://www.ncbi.nlm.nih.gov/projects/SNP/snp_ref.cgi?rs=rs770419845) in one Latino individual. It was not reported whether this individual was healthy, and the frequency was 0.017% (1/5789). The frequency of rs770419845 among the Chilean familial BC cases was 23-fold higher, at 0.4% (2/500). This result could be explained by ethnicity or by an association of this indel and the miR-17–92 cluster with familial BC susceptibility. The contemporary Chilean population stems from the admixture of Amerindian peoples with sixteenth- and seventeenth-century Spanish settlers. Nineteenth-century immigration by Germans, Italians, Arabs, and Croatians has had only a minor impact, with these groups accounting for ≤4% of the total population [18]. Therefore, the frequency of rs770419845 may be attributable to the genetic structure of this population.

This indel could be a pathogenic variant considering that a 1% cut-off value is recommended to separate benign polymorphic variants from mutations with pathogenic potential [19]. Similar criteria were established by Li et al. in 2016 [20]. Therefore, the frequency of the deletion (0.4%) in this cohort of cases does not rule out its potential as a pathogenic deletion. Another criterion for considering a mutation to be potentially pathogenic is that it is absent from controls (or is present at extremely low frequencies if recessive) in the Exome Sequence Project, 1000 Genomes Project, or the Exome Aggregation Consortium. Finally, a mutation can be considered potentially pathogenic if it corresponds to a short (2 or 6 bp) or large deletion that produces alterations. In this case, the delTTGGGC likely affects the structure folding in a deleterious manner. 

The indel was detected only in one of the 458 high-risk *BRCA1/2*-negative Chilean families. Nevertheless, the BC cases in the family, a mother and daughter, were both carriers of the deletion, and the daughter likely inherited the deletion from her mother. Importantly, the carriers of this mutation are two BC cases belonging to a BRCA 1/2-negative family that meets the criteria for a high-risk family, as there were two early-onset BC cases (48 and 37 years of age) and four gastric cancer cases in the family. BRCA 1/2 mutations have been recently described in gastric cancer [21]; thus, the gastric and BC cases in this family may be explained by this potentially pathogenic mutation. It is very unlikely that the mother and her daughter were both carriers of this deletion by chance; the minor allele frequency value (MAF) (0.002) is not compatible with a random event. Moreover, the American College of Medical Genetics and Genomics (AMCG) Standards and Guidelines [19] established that cosegregation of a variant with disease in more than one affected family member is a potential indicator of pathogenicity.

The first processing step in the biogenesis of a single miRNA relies on the RNase III enzyme Drosha, which releases the ~70-nucleotide miRNA-containing hairpins from the pri-miRNA. These precursor miRNAs (pre-miRNAs) are then exported out of the nucleus to the cytoplasm by Exportin 5/Ran GTPase and processed by another RNase III, Dicer, into mature ~22-nucleotide miRNAs. The miRNAs are subsequently incorporated into the RISC (RNA-induced silencing complex), which directs RNAi-mediated gene regulation by targeting a complementary mRNA [22,23,24,25]. miRNAs that reside in clusters represent a more complex case. The individual miRNAs within a cluster are processed with differential efficiency, despite being co-transcribed [26]. The delTTGGGC variant is located within the miR-17–92 cluster, which is among the best-characterized miRNA clusters. rs770419845 is positioned in the intergenic region, between pre-miR-17 and pre-miR-18a. This detail is important, given that the six miRNAs encoded by the cluster are often expressed at different levels, likely as a consequence of their processing efficiency, based in turn on their different levels of stability [12]. In the case of pri-miR-17–92a, as noted by Chakraborty et al., the transcript folds into a tertiary structure and autoregulates its processing [26]. Chaulk et al. and Chakraborty et al. established that tertiary RNA structure has a significant role in the biogenesis of cluster 17-92a, affecting the regulation of the individual miRNAs [25,26]. The miR-17-92a cluster adopts a compact globular structure where the 5′ region of the cluster folds on a 3′ core domain that contains the miR-19b and miR-92 hairpins. The internalized miRNAs are processed less efficiently than those on the surface of the structure. Disruption of the tertiary structure of the cluster exposes the buried miRNAs enabling more efficient processing and miRNA maturation. The increased processing of miR-92 results in an increased repression of a validated miR-92 target.

Chaulk et al. constructed a mutant of the miR-17-92a cluster and demonstrated an apparent correlation between Drosha processing efficiency and surface accessibility of the individual miRNA-containing hairpins [25]. Moreover, Chakraborty et al. designed two miniclusters, pri-miR-17-19a and pri-miR-20a-19a, and carried out expression studies in mammalian cells [26]. These authors reported that, as a control, the pre-miRNA level from the minicluster pri-miR-17-19a also undergoes processing to give significantly higher levels of all three pre-miRNAs relative to pri-miR-17-92a. Thus, a mere shuffling of discrete, pre-miRNA-containing hairpin domains is sufficient to alter the relative abundance of the processed pre-miRNAs. This scenario could be the case for the rs770419845 described in our short report. The cellular processing studies indicated structural differences between the native and shuffled transcript that impacted their processing, suggesting a more complex construction than a simplistic secondary structure model.

Modeling of the regions that included pre-miR-17 and -18a (Figure 2A) and the pri-miR of the complete cluster (Figure 2B) indicated that the secondary structure was altered when the deletion was present. A major change in the secondary structure at the level of the pre-miR-18a hairpin was also observed. The deletion is located between the genetic sequences for miR-17 and -18. The alterations to the secondary structure noted above are likely the consequence of the effect of the deletion on the tertiary structure, potentially affecting the efficiency of processing by Drosha. We also modeled the 17–92 cluster pri-miR sequence, randomly deleting six base pairs in any region of the sequence; the results of these deletions were less significant than the results of the deletion in the detected location, confirming the importance of the position of the deletion within the sequence of the 17–92a cluster.

Regarding the modeling algorithm for the region in which the deletion is located, the entropy of the position tends to zero. The positional entropy of an RNA region is zero when there is only one possible structure, excluding the possibility of dynamic equilibrium among multiple structures. In terms of the functionality of the RNA, both the specific structure and the dynamic stability of the region could be biologically important. Moreover, the algorithm predicts that the zone in which the deletion is located has a base pairing probability of 1 (the maximum possible value). Therefore, the deletion alters this pairing. Furthermore, in this region, the value of ΔG becomes more positive in the presence of the deletion, implying that the deletion affects the secondary structure, decreasing the stability of the pre-miR-17–pre-miR-18a sequence and the entire miR-17–92 cluster with respect to the wild-type structure. This set of variables supports the potential biological impact of the delTTGGGC deletion.

To our knowledge, this is the first report of this variant in association with human disease. With respect to BC susceptibility, this is also the first description of the variant in familial BC patients. There is no information available regarding the biological impact of this 6 bp variant, but it is possible that this deletion produces functional consequences. Specially, this variant may alter the biogenesis of the miR-17–92 cluster, thereby affecting expression levels of any of its six mature miRNAs. Functional studies are needed to confirm the functional impact of the indel delTTGGGC. 

## 4. Materials and Methods

### 4.1. Families

A total of 500 BC patients from 458 high-risk *BRCA1/2*-negative Chilean families were selected from Corporación Nacional del Cancer (CONAC) files. All index cases were tested for *BRCA1/2* mutations as previously described [27]. None of the families met strict criteria for other known syndromes involving BC, such as Li-Fraumeni, ataxia-telangiectasia, and Cowden disease.

Table 1 shows the characteristics of the families selected according to the inclusion criteria. All study families have self-reported Chilean ancestry dating from several generations, confirmed through extensive interviews with several members of each family from different generations. In the selected families, 13.1% (60/458) had bilateral BC cases, 11.8% (54/440) had both BC and OC cases, and 1.7% (8/458) had male BC cases. In the BC group, mean age at diagnosis was 42.8 years, and 81.4% (373/458) were diagnosed before 50 years of age.

The study was approved by the Institutional Review Board of the University of Chile, School of Medicine (Project code Number 1150117, 1 March 2015). Informed consent was obtained from all participants.

### 4.2. Controls

A healthy Chilean control sample (*n* = 480) was recruited from CONAC files. DNA samples were taken from unrelated individuals with no personal or familial history of cancer who provided consent for anonymous testing. These individuals were interviewed and informed as to the aims of the study. DNA samples were obtained according to all ethical and legal requirements. The control sample was matched to the case population for age and socioeconomic strata.

### 4.3. Mutation Analysis

Genomic DNA was extracted from peripheral blood lymphocytes of BC cases and controls. Samples were obtained as described by Chomczynski and Sacchi [28].

### 4.4. Pre-miR-17 Complete Sequence Analysis

This analysis was performed in 99 of the 500 cases from 458 families with (a) ≤3 family members with BC and/or OC (83.8%) and (b) index cases with early-onset BC (≤35 years) (16.2%). The whole coding sequence and pre-miR-17 sequence boundaries were amplified by polymerase chain reaction (PCR). Primers were designed with Primer3 v.0.4.0 (Whitehead Institute for Biomedical Research, Cambridge, UK; Boston, MA, USA) [29]. Sequencing was performed in an ABI 3730xl automated fluorescence-based sequencer and BigDye v.3.1 terminator system (Applied Biosystems, Foster City, CA, USA).

### 4.5. Pre-miR-17 rs770419845 Analysis

Genotyping of rs770419845 was performed using DNA fragment analysis by capillary electrophoresis in an ABI 3730xl DNA analyzer (Applied Biosystems, Foster City, CA, USA) for BC cases and controls. The reaction was performed in a 50 µL final volume containing 100 ng of genomic DNA, 1.5 mM MgCl_2_, 0.2 mM each dNTP, 10 pmol primer (forward primer [5′-TCAAAGTGCTTACAGTGCAGGT-3′]) and reverse primer [5′-GGAGCACTTAGGGCAGTAGATG-3′]), and 1.5 UI GoTaq Flexi DNA polymerase (Promega Corporation, Madison, WI, USA). The Thermal Cycler program was 95 °C × 2 min, ([95 °C × 30 s, 60 °C × 30 s, 72 °C × 30 s] × 30 cycles]), 72 °C × 5 min, and 4 °C × 5 min. Forward primers were marked with 6′FAM (Fluorescein) (Integrated DNA Technologies, San Diego, CA, USA).

### 4.6. Bioinformatic Analysis

In silico analyses to predict the effect of rs770419845 on the physicochemical parameters and secondary structure of pre-miR-17 RNA and the whole miR-17–92 cluster were performed using RNAfold from the ViennaRNA Web Service (Available online: http://rna.tbi.univie.ac.at/) [13].

## 5. Conclusions

To our knowledge, this is the first report of this variant in association with BC. No information is available regarding the biological impact of delTTGGGC. This deletion could alter the biogenesis of the miR-17–92 cluster and thus affect the expression levels of any of its six mature miRNAs. Based on bioinformatic analyses, the location of the deletion, and its low frequency, we presume rs770419845 may be pathogenic variant. Functional studies are needed to determine the biological impact of the delTTGGGC indel and support this hypothesis.

## Figures and Tables

**Table 1:** Inclusion criteria for families.

Inclusion Criteria	Families: *n* (%)
≥3 family members with breast and/or ovarian cancer	139 (30.4%)
2 family members with breast and/or ovarian cancer	155 (33.8%)
Single affected individual with breast cancer, diagnosed at ≤35 years of age	81 (17.7%)
Single affected individual with breast cancer, diagnosed at 36–50 years of age	83 (18.1%)
Total	458 (100%)
